# Annular Elastolytic Giant Cell Granuloma

**DOI:** 10.7759/cureus.11456

**Published:** 2020-11-12

**Authors:** Anuja Mahesh Mistry, Rutul Patel, Mahesh Mistry, Varna Menon

**Affiliations:** 1 Internal Medicine, Smt Nathiba Hargovandas Lakhmichand Municipal Medical College, Ahmedabad, IND; 2 Internal Medicine, Smt. Nathiba Hargovandas Lakhmichand Municipal Medical College, Ahmedabad, IND; 3 Dermatology and Venereology, Saham Hospital, Saham, OMN; 4 Pathology and Laboratory Medicine, Sohar Hospital, Sohar, OMN

**Keywords:** elastolysis, actinic granuloma, annular, giant cell, sun exposure

## Abstract

Annular elastolytic giant cell granuloma (AEGCG) is a rare, often self-limiting chronic inflammatory disorder mostly occurring in the sun-exposed areas such as the dorsum of hands, extensor surfaces of arms, face, anterior neck, and upper chest. The pathognomonic histological findings include the presence of numerous granulomas associated with loss of elastic fibers that appear to be ingested by multi-nucleated giant cells. Here, we present a case of a 56-year-old woman with multiple, anatomically variable erythematous lesions- annular and papular, mainly in the upper body. The clinical presentation and histopathologic findings support our diagnosis.

## Introduction

AEGCG is a chronic inflammatory dermatological disease that presents in adults aged between 35 to 75 years and is identified by the presence of raised annular lesions mainly on the sun-exposed skin [1]. The pathogenesis of the disease remains a question for discussion, but the role of inflammation in the process has been well established [2]. Due to the physical similarities between AEGCG and granuloma annulare, confirmation of the diagnosis with skin biopsy becomes imperative. Several pharmacotherapy regimens have been tried, which include retinoids, corticosteroids, hydroxychloroquine, methotrexate, cyclosporine, and dapsone [3-8], along with sun-protective measures. We present a case of a 56-year-old woman with a long-standing history of progressive skin lesions on the forehead and upper body, associated with mild pruritus. She reports a past medical history of hypertension, well-controlled on medication. 

## Case presentation

**Figure 1:**
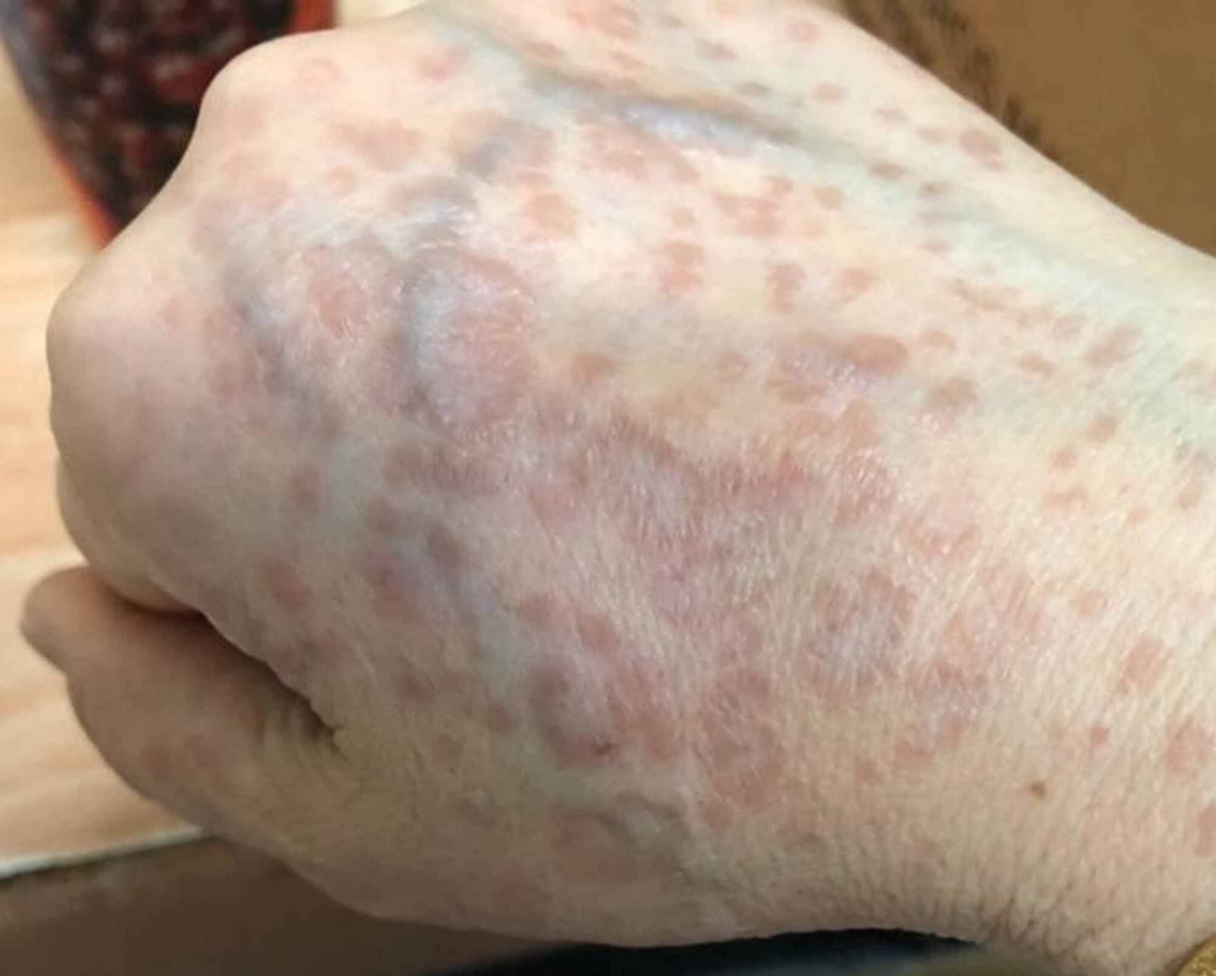
Annular lesions on the dorsum of hand

A 56-year-old woman, presented with insidious onset, gradually evolving, multiple erythematous papules on the dorsum of the left hand for 6 months. The lesion began with a single reddish-brown papule on the left dorsal hand with mild pruritus, consequently leading to multiple such lesions forming an annular pattern that gradually progressed to involve the left arm, anterior neck and chest, and right upper limb over a period of 5-6 months (Figure 1). The patient noted that the lesions worsened after sun exposure, causing aggravation of her condition in summer. Associated co-morbidities included hypertension, well-controlled with Amlodipine 5 mg, once daily.

**Figure 2:**
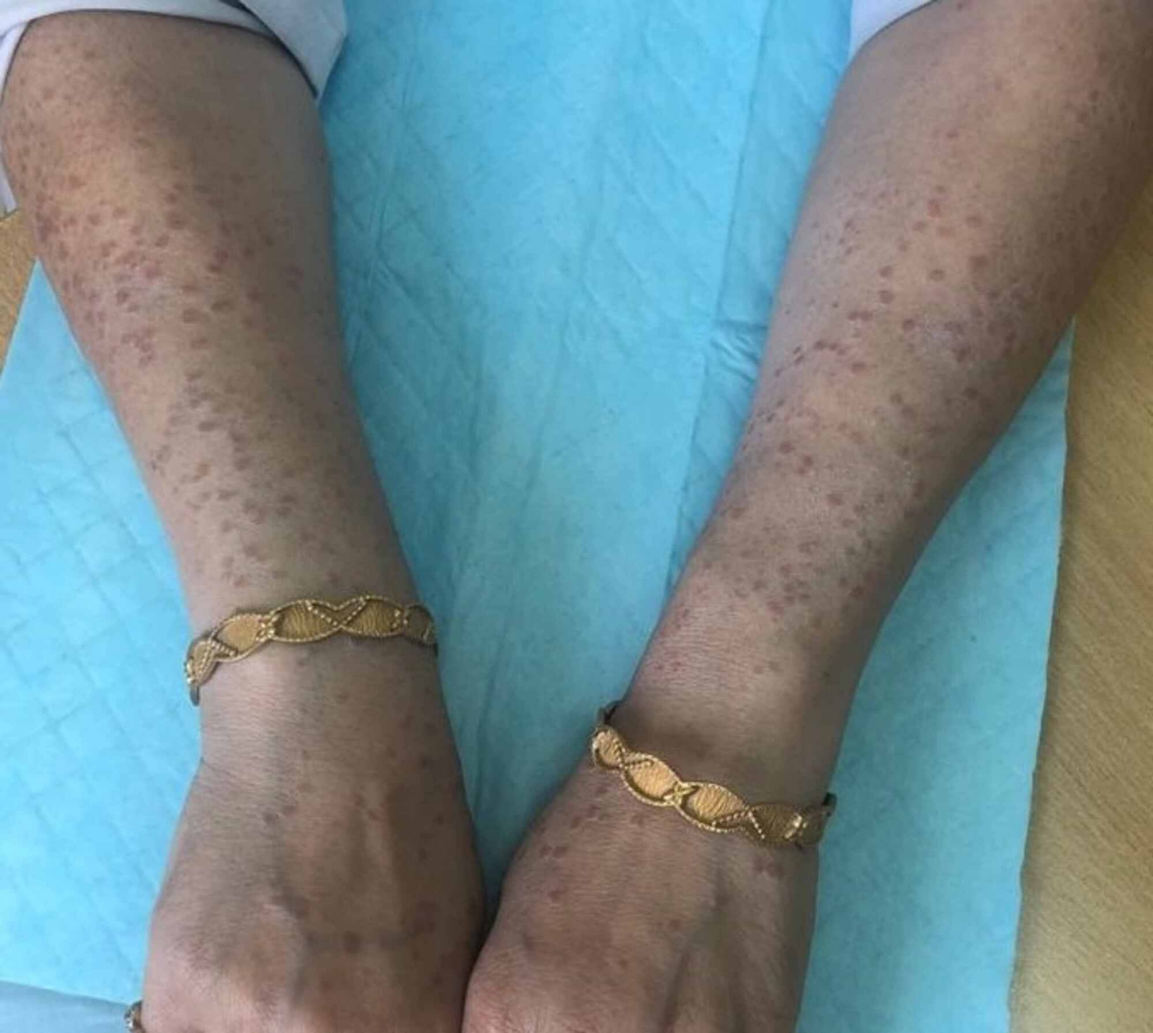
Erythematous papular lesions on bilateral forearms

**Figure 3:**
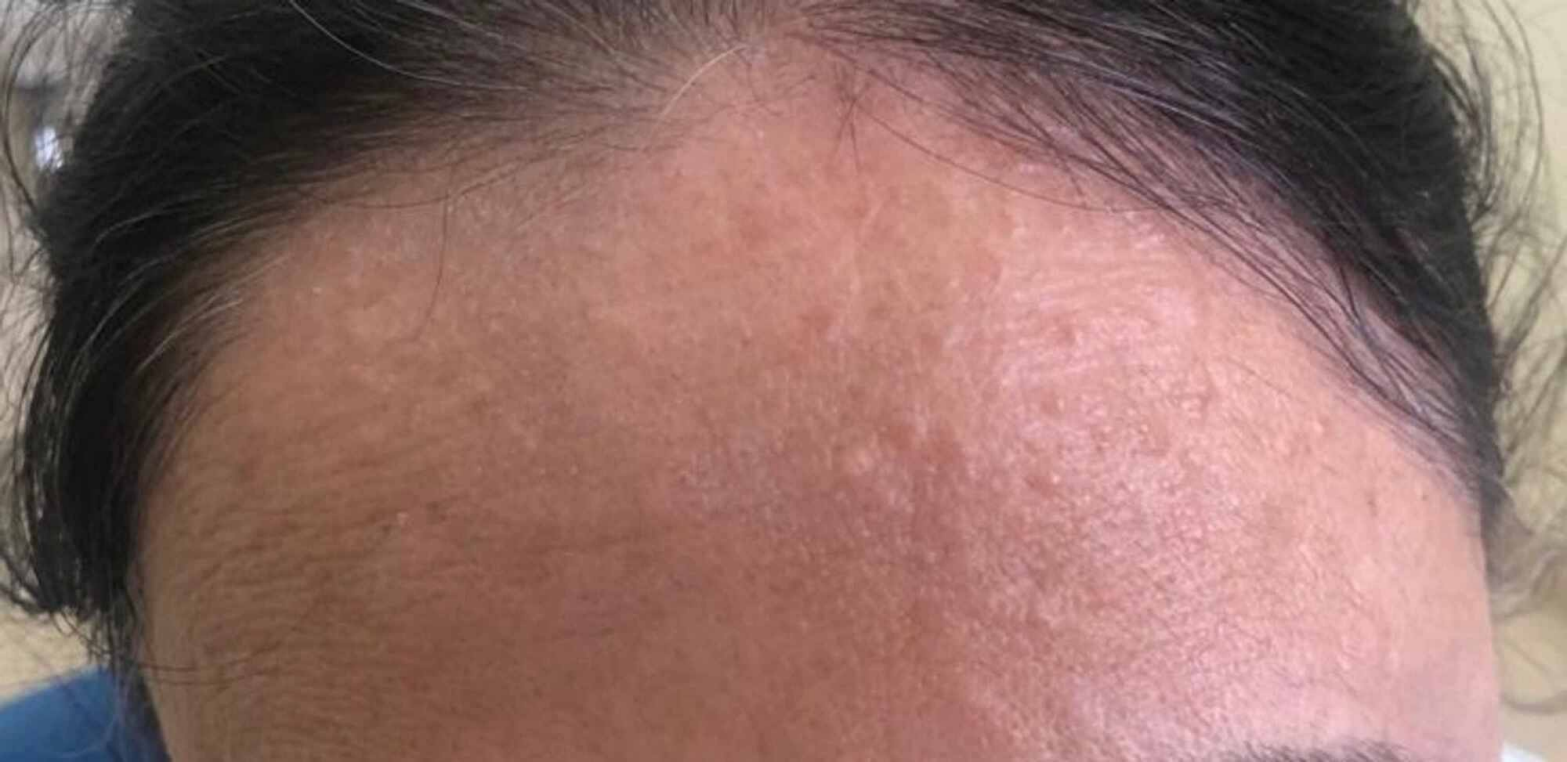
Papular lesions on forehead

On physical examination, multiple erythematous papules and plaques, reddish-brown to tan-colored, measuring approximately 2 mm to 5 mm were noted on bilateral upper limbs, forehead and “V” area around the neck, some of the lesions appeared annular (Figure 2, 3). Lesions were not warm, non-tender, not associated with scales/blisters/scars/drainage of fluid.

Initial routine investigations including complete blood count (CBC), erythrocyte sedimentation rate, Serum thyroid-stimulating hormone (TSH), basal metabolic panel, liver function tests, and serum anti-nuclear antibody (ANA) titer were within normal limits or negative.

A preliminary diagnosis of photodermatitis was made, and the patient was started on topical Betamethasone and Mometasone, and was advised on preventative measures-protective clothing and the use of sunscreens and umbrella with Ultraviolet (UV) protection. On a follow-up visit three months later, only negligible clinical improvement was observed.

**Figure 4:**
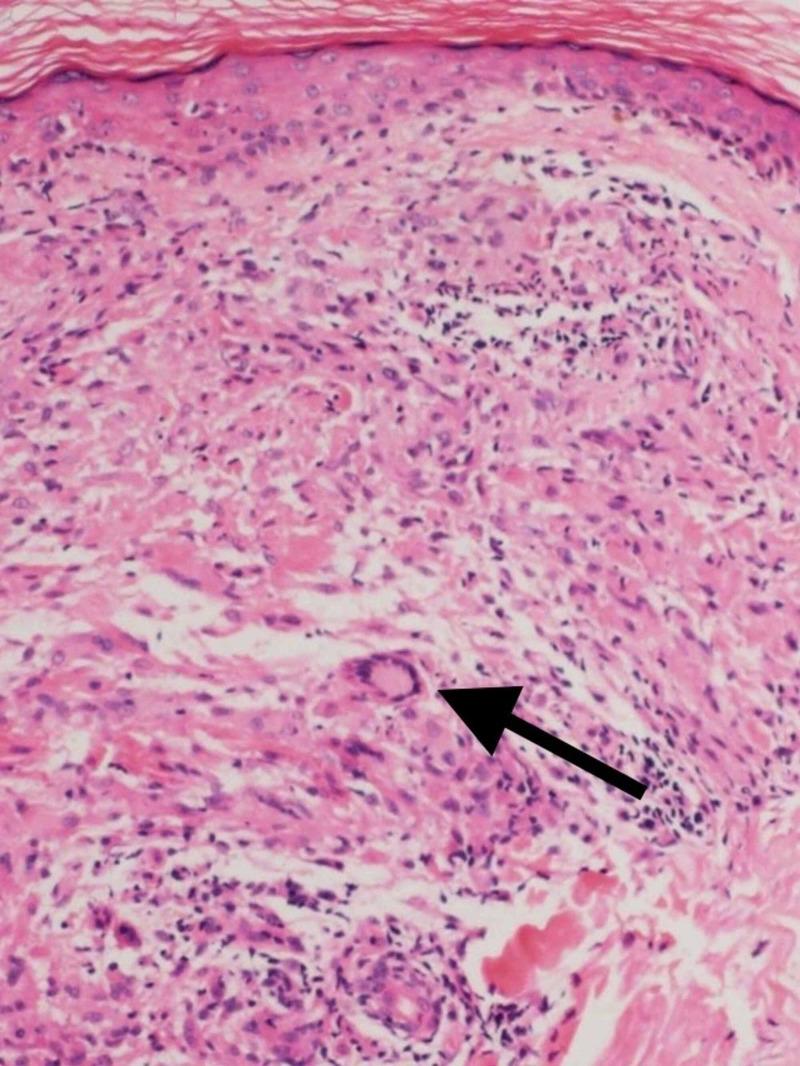
H&E 10X magnification showing multiple granulomas composed of histiocytes and giant cells Arrow head points to the multi-nucleated giant cell within the dermis

**Figure 5:**
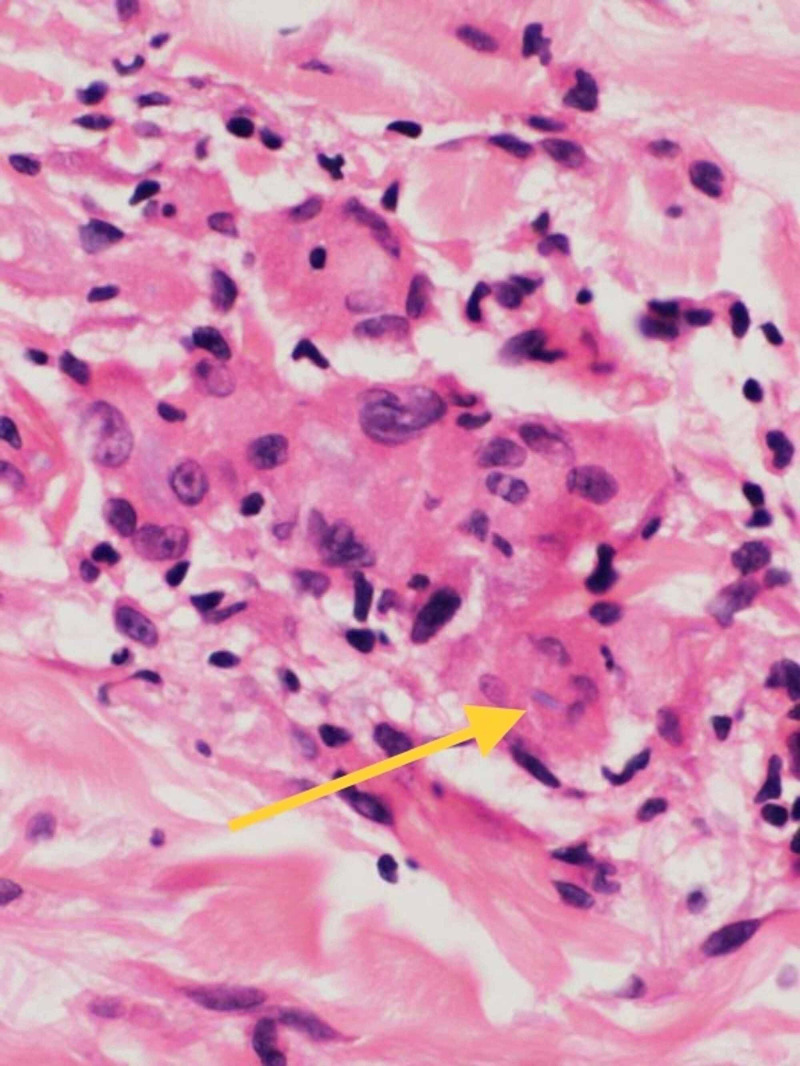
H&E 40X magnification showing giant cells with elastophagocytosis Arrowhead points to the ingested fibers seen within the giant cell

**Figure 6:**
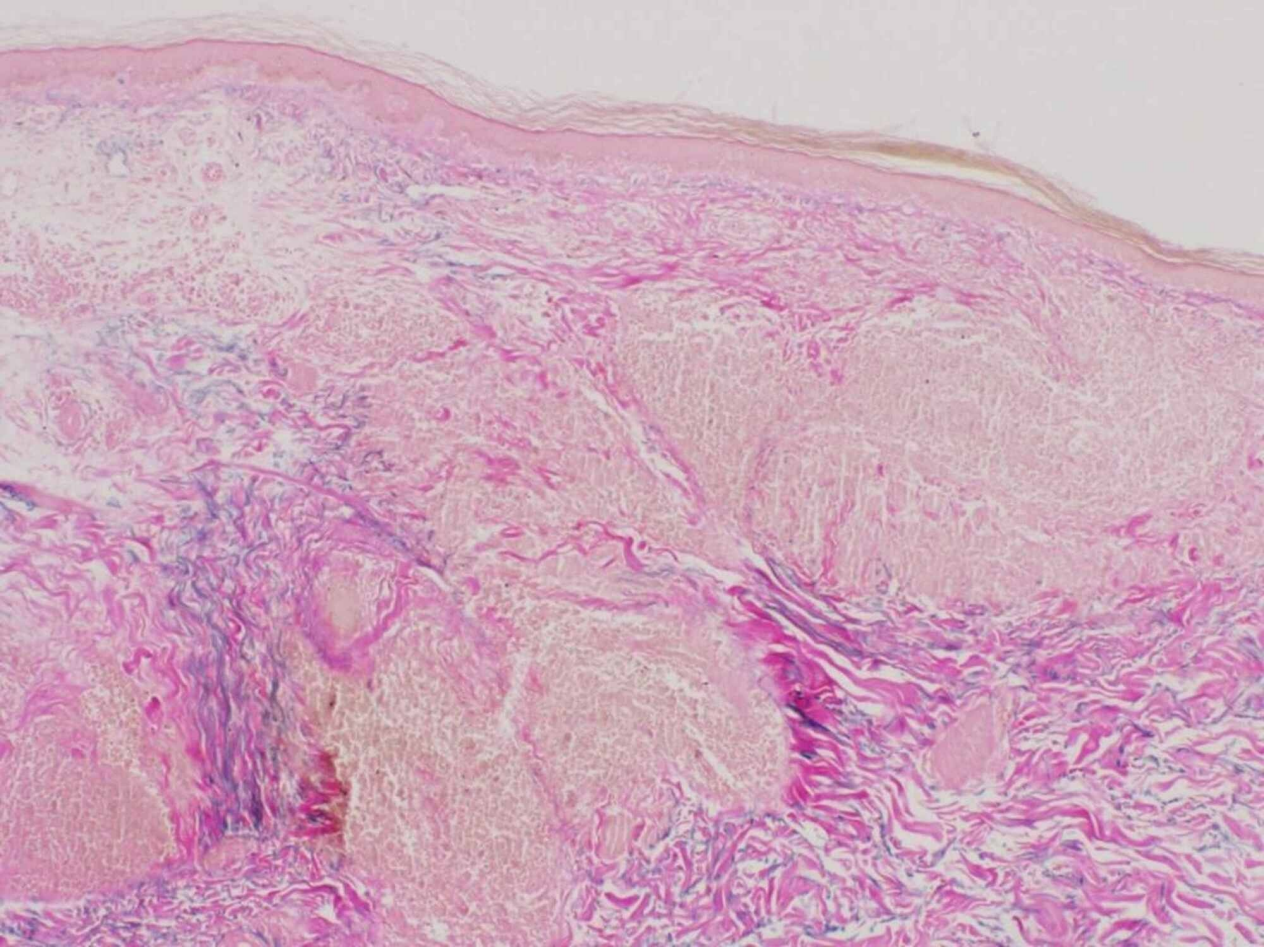
eVG stain 10X magnification reveals granulomas with complete loss of elastic fibers associated with preserved abundance of elastic fibers at the periphery of granuloma eVG: elastin Verhoeff Van Gieson

**Figure 7:**
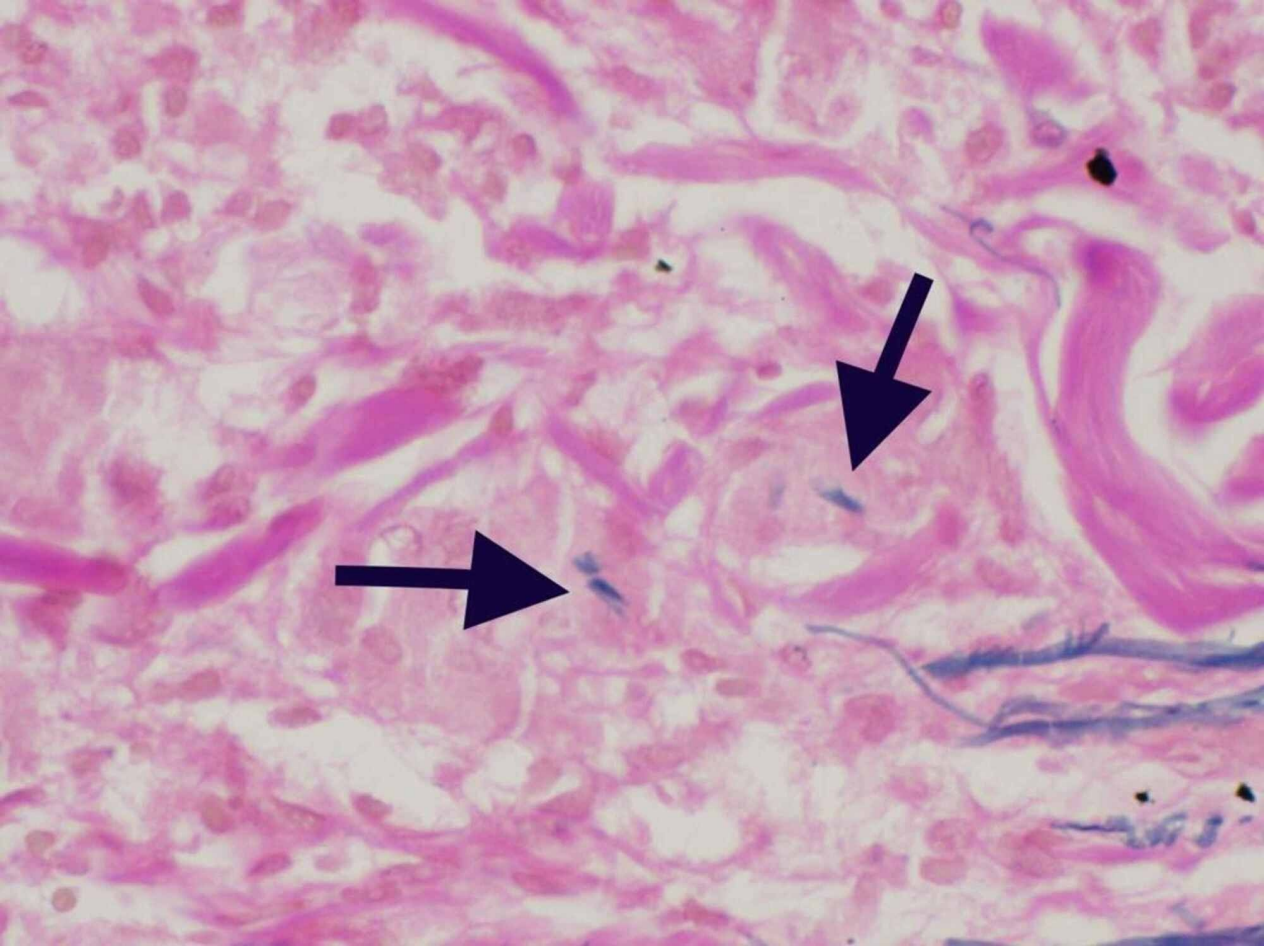
eVG stain 40X magnification showing giant cells with elastophagocytosis eVG: elastin Verhoeff Van Gieson; Arrowheads pointing to the ingested fibers within the giant cells

Punch biopsy taken from right lateral forearm revealed multiple foci of granulomatous infiltrate composed of epithelioid mono- and multi-nucleated histiocytes and giant cells in the superficial dermis with elastolysis. The periphery of the granulomas highlighted lymphocytes and plasma cells (Figure 4, 5). Special staining for elastic fibers (Verhoeff Van Gieson stain) revealed complete loss of elastic tissue in the granulomatous zone along with fragmented elastic fibers within giant cells (Figure 6, 7). Special staining for Acid Fast Bacilli (AFB) and Periodic Acid Schiff (PAS) were negative for micro-organisms, and Alcian Blue PAS (AB PAS) for stromal mucin was negative as well.

**Figure 8:**
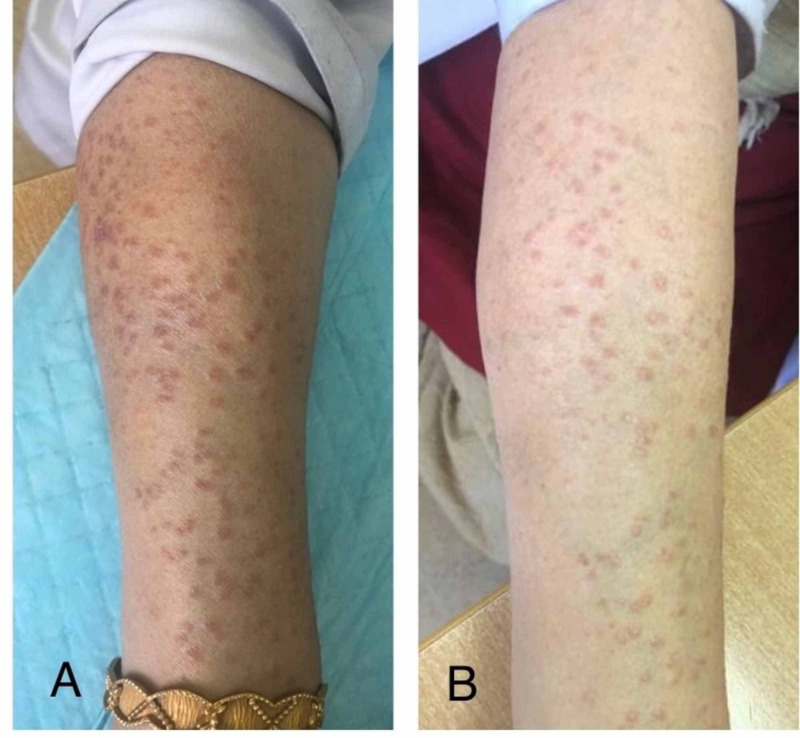
Comparative pictures of before (A) and after (B) three months of treatment with systemic corticosteroids

After histopathologic confirmation of the diagnosis, several treatment options were discussed. Hydroxychloroquine, methotrexate, and cyclosporine were refused by the patient due to the risk of systemic toxicity. Therefore, the patient was initiated on oral dexamethasone in a tapering dose of 4mg daily x 10 days, followed by 2mg daily x next 10 days, followed by 2mg every alternate day x 10 days, further followed by 2mg twice-weekly doses. This regimen was continued for several months to maintain remission. Figure 8 shows improvement after treatment with oral Dexamethasone.

**Figure 9:**
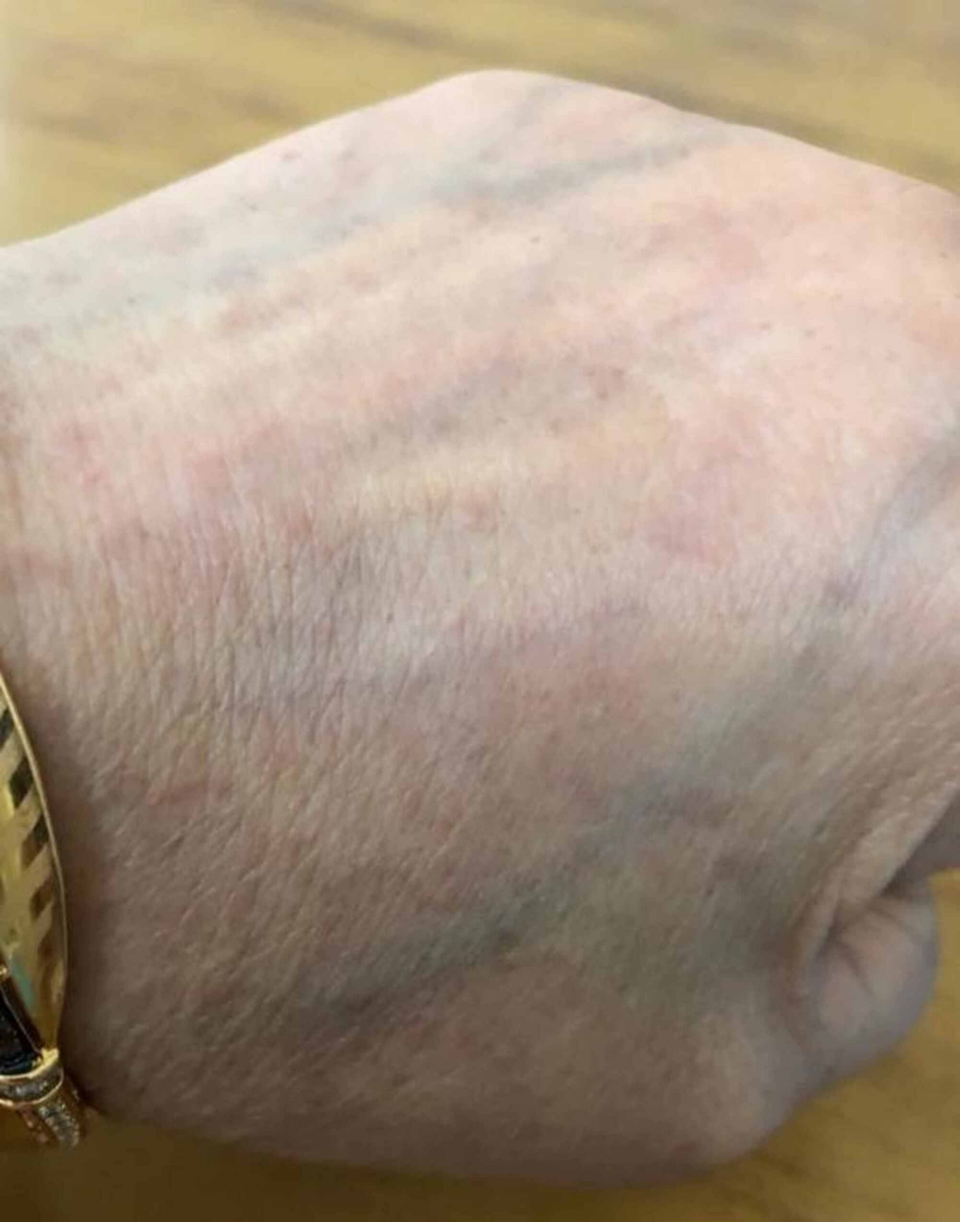
Left dorsal hand showing healing lesions, 8 months since the onset of symptoms

**Figure 10:**
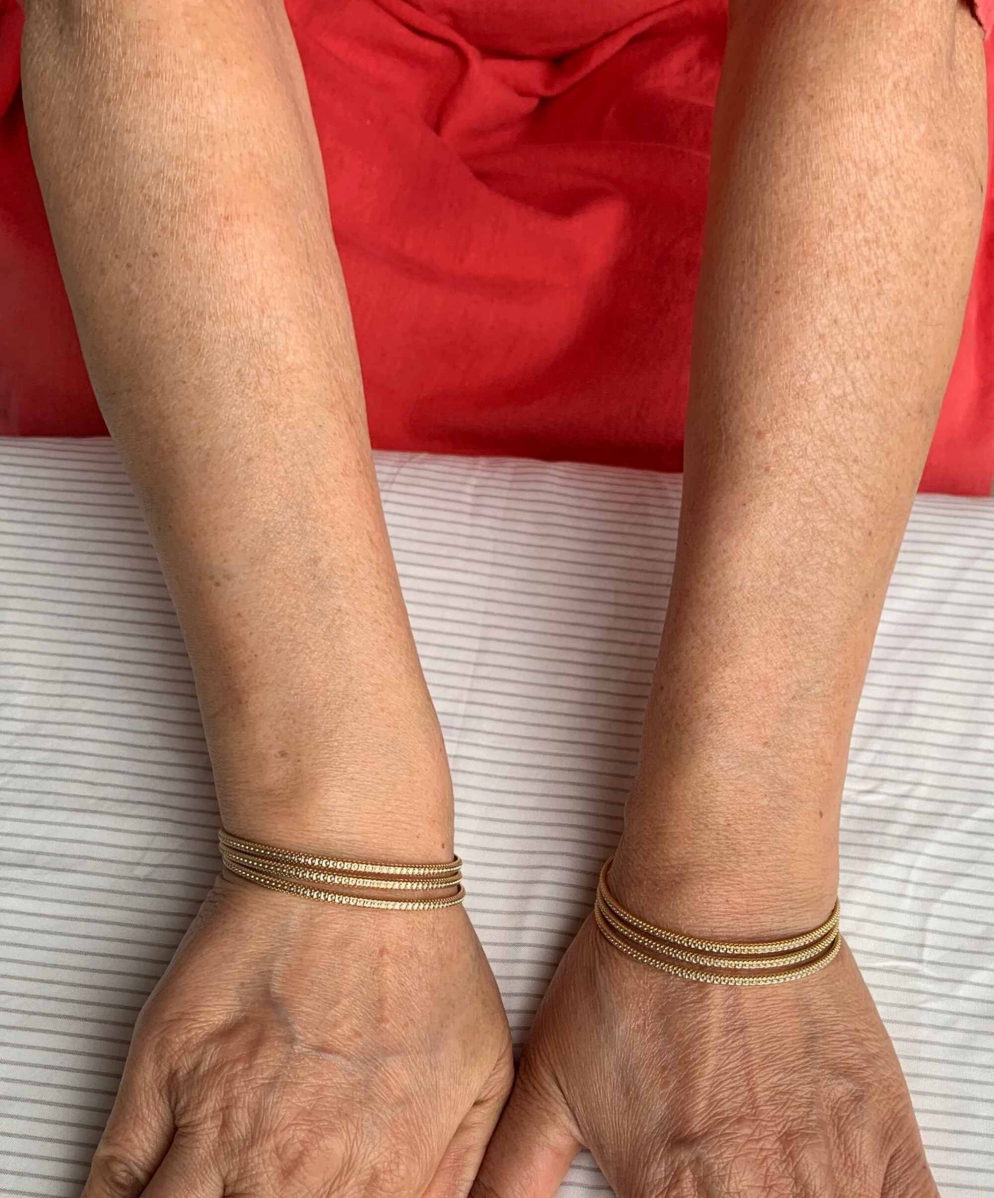
Bilateral hands showing complete healing, one and a half years since onset of symptoms

At one of the follow-up visits, approximately 2 months since initiation of oral dexamethasone, random blood sugar was noted to be elevated, and CBC highlighted neutrophilia, which mandated discontinuation of systemic corticosteroid that eventually led to an exacerbation of her skin lesions. Therefore, she was re-initiated on the same regimen with behavioral modifications such as weight loss and strict control of blood sugar level. Figure 9 and Figure 10 reveal the stages of healing at different points in time.

## Discussion

Actinic granuloma, also called as annular elastolytic giant cell granuloma, was first narrated by O’Brien in 1975 [1]. It refers to a rare, idiopathic inflammatory skin disease of middle-aged adults, characterized by annular plaques mainly found on sun-exposed skin [9]. Pathogenesis has not been clearly understood and is highly debatable. Several postulations have been put forward, a few of which include-

1. O’Brien’s Actinic hypothesis: Solar radiation is the initial trigger that selectively causes damage to the elastic tissue in the upper and mid-dermis. This degenerated tissue then becomes a target for an auto-immune cell-mediated response (predominantly CD4+ cells), that attempts to repair the damaged skin, but eventually leads to granulomatous inflammation instead. This theory is consistent with most of the findings seen in our patient- lesions in sun-exposed skin, improvement of lesions with stringent avoidance of sun exposure, fragmented elastic fibers ingested by giant cells, and presence of lymphocytes. However, this theory was adamantly opposed by Ragaz and Ackerman who re-instated that these granulomas were merely an anatomical variant of granuloma annulare [2,10].

2. Inflammatory theory- arguably suggests that the elastic fiber destruction is caused by granulomatous inflammation itself, implying that inflammation is the inciting event; rather than actinic radiation [10]. This theory is further strengthened by a study performed by Kiken et al. where a 4-day solar provocation testing failed to induce new actinic lesions in a patient with pre-existing actinic granuloma [11].

Photo-distributed erythematous annular plaques surrounding a pale/ hypo-pigmented center and elevated borders are seen on examination [3]. The number of lesions may vary from a single large lesion to clusters of thousands of smaller lesions [4,9]. Contrary to the typical annular pattern, our patient presented with papular, flat raised, multiple reddish-brown to tan-colored, non-coalescing lesions widely distributed across sun-exposed skin, with a few lesions in areas not associated with solar exposure; only a few annular lesions were reported. As per O’Brien, three distinctive histologic zones can be identified [3,10,12,13] which consist of a central zone with sub-total to complete absence of elastic fibers, peripheral zone composed of an increased amount of elastotic material best seen with Verhoff Van Gieson stain, and a zone of raised border showing granulomatous infiltrate with histiocytes either in palisading fashion or interstitially within collagen bundles. The four histopathologic variants proposed by O’Brien are [10] :

- Giant cell variant: As seen in our patient, consists of large, foreign body giant cells, with few histiocytes and other inflammatory cells that secrete elastase, causing widespread elastosis.

- Necrobiotic variant (vascular variant): believed to occur due to ischemia of sun-damaged skin, showing actinic arteriopathy with single/ multiple necrobiotic foci and palisading giant cells.

- Sarcoid variant: has resemblance with the sarcoid (tuberculoid) system with fibrocellular infiltrate and clusters of histiocytes and sarcoid arteritis.

- Histiocytic variant: slower, less florid form; very few giant cells, if present, are smaller; histiocytes are scattered near elastic fibers.

As per a study by Raposo et al., a slight male predominance was noted [14]; however, an integrated data collection concludes male: female ratio of 1.2:1 to 2:1 [4-7]. The most common age of onset is between 35 to 75 years [6,7]. The most commonly reported risk factor is intense sun exposure/tanning beds and is more commonly seen in fair-skinned individuals [15] residing in a sunny climate. Other possible predisposing conditions and associations include diabetes mellitus (DM), sarcoidosis [7], giant cell arteritis [5], polymyalgia rheumatica, polychondritis, vitiligo [3,9], anemia, leukopenia, hepatitis C, focal segmental glomerulosclerosis, Hashimoto's thyroiditis [16], erythema nodosum [17] and protoporphyria [18]. A list of possible differentials for our patient included photodermatoses (lack of suggestive histopathologic findings in this patient rules it out), granuloma annulare, necrobiosis (lack of evidence of DM), drug-induced photosensitivity (lack of consumption of associated medications), dermal manifestations of auto-immune disorders, such as systemic lupus erythematosus (negative ANA titer). Al Hoqail et al. and a few other studies concluded the following from their studies [3,8,19,20] :

**Table 1:** Differences between AEGCG and GA AEGCG: Annular elastolytic giant cell granuloma; GA : Granuloma annulare; MNGC : Multi-nucleated giant cells

Pertaining histopathologic findings	AEGCG	GA
Granuloma type	Non-palisading, interstitial, sarcoidal	Palisading
Granuloma location	Superficial dermis only	Superficial and/or deep dermis
MNGC	Giant cells with as many as 12 nuclei	Giant cells contain +/- 3 nuclei
Presence of mucin	Absent	Present
Necrobiosis	Absent	Present
Distribution of lesions	Mostly photosensitive	Variable
Elastotic material	Abundant outside granuloma, less within, associated with scarring within the granuloma	Moderate, within and around granuloma, no scarring

Various modalities that have been employed for treatment are intralesional/systemic corticosteroids, hydroxychloroquine [8], cyclosporine, azathioprine, psoralen ultraviolet A (PUVA) rays, topical calcineurin inhibitors (pimecrolimus, tacrolimus) [5,7]. Others include dapsone, clofazimine, methotrexate, systemic retinoids [3-5]. Our patient was followed up for a period of one and a half years and the most effective treatments for her were strict adherence to avoiding sun exposure, and intermittent doses of systemic corticosteroids.

## Conclusions

Actinic Granuloma(AEGCG), being a rare dermatogical condition, necessitates the need for reporting more cases and research to further widen our medical perspective, which is the reason that we believe this case is worth reporting. The following information in this patient should be taken into consideration: firstly, her profession (doctor) was not precisely associated with sun exposure, which is the most commonly associated risk factor. Secondly, instead of characteristic annular lesions, most of her lesions were papular and pruritic. Lastly, despite the association with solar-exposure, few lesions appeared on non-sun exposed skin.
